# Selective mediation of ovarian cancer SKOV3 cells death by pristine carbon quantum dots/Cu_2_O composite through targeting matrix metalloproteinases, angiogenic cytokines and cytoskeleton

**DOI:** 10.1186/s12951-021-00813-8

**Published:** 2021-03-04

**Authors:** Daomei Chen, Bin Li, Tao Lei, Di Na, Minfang Nie, Yepeng Yang, Congjia, Xie, Zijuan He, Jiaqiang Wang

**Affiliations:** 1 National Center for International Research On Photoelectric and Energy Materials, School of Materials and Energy, Yunnan University, Kunming, 650091 People’s Republic of China; 2 Key Laboratory of Medicinal Chemistry for Natural Resource, Ministry of Education, Yunnan University, Kunming, 650091 People’s Republic of China; 3 School of Chemical Sciences & Technology, Yunnan University, Kunming, 650091 People’s Republic of China

**Keywords:** Ovarian cancer SKOV3 cells, Cellular microenvironment, Matrix metalloproteinases, Cytoskeleton, Angiogenesis, MMP-2/9, VEGFR2, F-actin

## Abstract

It was shown that some nanomaterials may have anticancer properties, but lack of selectivity is one of challenges, let alone selective suppression of cancer growth by regulating the cellular microenvironment. Herein, we demonstrated for the first time that carbon quantum dots/Cu_2_O composite (CQDs/Cu_2_O) selectively inhibited ovarian cancer SKOV3 cells by targeting cellular microenvironment, such as matrix metalloproteinases, angiogenic cytokines and cytoskeleton. The result was showed CQDs/Cu_2_O possessed anticancer properties against SKOV3 cells with IC_50_ = 0.85 μg mL^−1^, which was approximately threefold lower than other tested cancer cells and approximately 12-fold lower than normal cells. Compared with popular anticancer drugs, the IC_50_ of CQDs/Cu_2_O was approximately 114-fold and 75-fold lower than the IC_50_ of commercial artesunate (ART) and oxaliplatin (OXA). Furthermore, CQDs/Cu_2_O possessed the ability to decrease the expression of MMP-2/9 and induced alterations in the cytoskeleton of SKOV3 cells by disruption of F-actin. It also exhibited stronger antiangiogenic effects than commercial antiangiogenic inhibitor (SU5416) through down-regulating the expression of VEGFR2. In addition, CQDs/Cu_2_O has a vital function on transcriptional regulation of multiple genes in SKOV3 cells, where 495 genes were up-regulated and 756 genes were down-regulated. It is worth noting that CQDs/Cu_2_O also regulated angiogenesis-related genes in SKOV3 cells, such as Maspin and TSP1 gene, to suppress angiogenesis. Therefore, CQDs/Cu_2_O selectively mediated of ovarian cancer SKOV3 cells death mainly through decreasing the expression of MMP-2, MMP-9, F-actin, and VEGFR2, meanwhile CQDs/Cu_2_O caused apoptosis of SKOV3 via S phase cell cycle arrest. These findings reveal a new application for the use of CQDs/Cu_2_O composite as potential therapeutic interventions in ovarian cancer SKOV3 cells.

**Supplementary Information:**

The online version contains supplementary material available at 10.1186/s12951-021-00813-8.

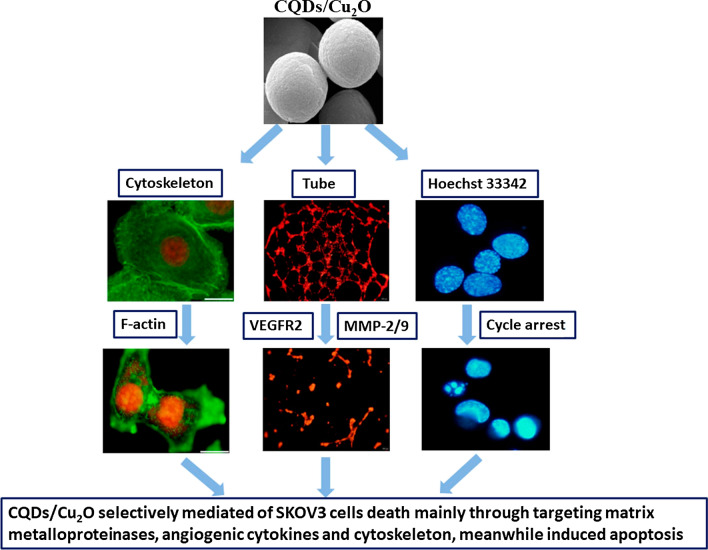

## Introduction

In gynecological malignancies, ovarian cancer is the one of the leading causes of death [1, 2]. Although combination of platinum and a taxane-containing agent remains the major treatment method after surgical resection, most patients ultimately succumb to the disorder because of the limited effects of the treatment on cancer growth, relapse, and drug resistance [3, 4]. Consequently, there is an urgent need to exploit more effective treatment means to treat ovarian cancer and delay or prevent recurrences [5].

Compared with traditional treatments, nanomaterials offer new opportunities for the development of diagnostic and therapeutic tools for cancer and other diseases [6–8]. Ag, Au, ZnO, TiO_2_, As_2_O_3_, graphene oxide-silver nanoparticle, and iron core-gold shell nanoparticles have been reported to cause the apoptosis of cancer cells [9–13]. It was shown that some nanomaterials may have anticancer properties, mainly due to toxicity of the nanomaterials, but there is still a lack of nanomaterials that selectively distinguish cancer cells from normal cells.

More and more evidences indicated that cancer progression is closely related to the tumour microenvironment, including the extracellular matrix (ECM) deregulation, blood vessels expanding and immune response suppression [14]. Matrix metalloproteinases (MMPs) are a family of enzymes, have the ability to proteolytically degrade various components of ECM, participate in remodeling of basement membranes and contributed to angiogenesis [14]. The decrease of MMPs expression or enzymatic activities is considered to be the main factor for the inhibition potential of migration and angiogenesis. Angiogenesis, supplies oxygen and nutrients to actively proliferating cancer cells, provides advantages for cancer cells growth, invasion and metastasis [15, 16]. Numerous signalling molecules and pathways associated with angiogenic responses in the tumour microenvironment, play major roles in cancer cell growth and metastasis [17, 18]. Therefore, developing MMPs and angiogenesis inhibitor has become an effective tumor treatment strategy. Most current reports focus on targeting the vascular endothelial growth factor (VEGF). For example, gadolinium metallofullerenol nanoparticles, AgNPs, [19] and hollow mesoporous carbon nanocapsules (HMCNs) [20] have been reported to regulate tumour angiogenesis by VEGF pathway. We found that Fe-MIL-101 have an anti-angiogenesis potential utility by reducing MMP-2/9 expression [21].

In addition, the regulation of cytoskeleton plays a key role in the process of metastasis in cancer cells [22]. Actin, as an important part of cytoskeleton, has the function of maintaining the communication between cytoplasmic proteins and transmembrane, keeping mechanical strength, and regulating cells locomotion [22]. Recently, several studies have reported the effects of some nanomaterials on actin cytoskeleton in cancer cells. For example, carbon nanomaterial [23], curcumin analog MHMD [24], grapheneoxide nanosheets [25], AgNPs [26], and ZnONPs [27] affected F-actin cytoskeleton through targeting or disruption of F-actin. It has been reported the metal–organic frameworks (IRMOF-3) possesses the ability to disrupt F-actin and tubulin, blocking the rat pheochromocytoma cell division [28]. Recently, we found Cu-MOF has an intrinsic activity of protease-mimicking [29] and disrupts of F-actin in ovarian cancer cells, leading the mitotic catastrophe [30].

Carbon quantum dots (CQDs) are currently eliciting much attention in cancer therapy due to outstanding properties including biocompatibility, low cytotoxicity, water solubility and unique photoluminescence [31]. CQDs and CQDs-based composites can be used for anti-cancer by phototherapy and radiotherapy. For instance, CQDs inhibited human breast cancer cells MCF-7 and MDA-MB-231 by photodynamic therapy triggering the formation of singlet oxygen species [32]. It was reported CQDs could be used as photosensitizers to destroy buried tumors in photodynamic therapy. C–Ag-PEG CQDs inhibited Du145 cells by free radicals in radiotherapy, which reduced the damage of normal cells and increased therapeutic selectivity [33]. It has been reported CQDs can selectively target cancer cells through regulated the expression of major angiogenic cytokines, such as VEGF, FGF, and VEGFR2 [34]The results were shown that the angiogenesis inhibition rate of 100 μg CQDs was less than 40%. It is an effective strategy for tumor therapy to develop carbon quantum dot composite materials, making use of its synergistic effect to improve the anti-angiogenesis activity.

Cu_2_O NPs has been extensively researched to elucidate their significance in cancer therapy. Cu_2_O NPs significantly reduce the growth and metastasis of melanoma, improve the viability of tumor-bearing mice, and induce the mitochondrion-mediated apoptosis of cancer cells [35]. Cu_2_O NPs was more sensitive to rapidly proliferating HeLa cancer cells than normal human kidney 293 T cells, indicating Cu_2_O NPs exert distinct effects on different cells [36]. Since one of the biggest challenges of chemotherapy is that chemotherapy drugs do not effectively distinguish between tumor and normal cells, differential cytotoxicity is very important [36].

Gene expression experiments, as a method for detection of disease markers, has been used to assessment the toxicity mechanism of nanomaterials [37]. For example, Ag nanoparticles involved regulating gene expression, including oxidative phosphorylation gene, protein synthesis gene, vascular endothelial growth factor-A (VEGFA) gene and fibroblast growth factor2 (FGF2) gene in different cell lines [38, 39]. Single-walled carbon nanotubes also regulated gene expression of relative root growth, influence [40]. Melittin-loaded ZIF8 nanoparticles was found to induce A549 cells apoptosis through regulating the expression of 3383 genes [41]. Recently, we reported that Cu-MOF (HKUST-1) induced mitotic catastrophe through destruction of actin cytoskeleton and changes of gene expression in SKOV3 cells [30].

On the other hand, the proteases naturally expressed by living organisms participate in all stages of tumor progression. In our previous study, we found that the CQDs/Cu_2_O composite possessed an intrinsic protease-mimicking activity for hydrolyzing proteins under physiological conditions, ultimately exhibiting a surprisingly higher catalytic activity than Cu_2_O and CQDs [42]. Based on the above findings, we investigated the inhibitory mechanism of CQDs/Cu_2_O composite on human ovarian cancer SKOV3 cells. It will be attractive to elucidative the potential roles of the CQDs/Cu_2_O composite in the regulation of cancer-related proteins. Furthermore, only a few nanoparticles have been reported to interact with the tumour microenvironment. As far as we know, this is the first attempt to reveal CQDs/Cu_2_O composite mediates tumor microenvironment and cytoskeleton.

## Experiment section

### Reagents

CuSO_4_, poly-vinylpyrrolidone, and C_6_H_12_O_6_ were obtained from Shanghai Tian Scientific Co. Ltd. Cell culture medium (Dulbecco's modified eagle’s medium, DMEM) were from Hyclone Laboratories. Fetal bovine serum (FBS) were got from Gibico. Acridine orange, ethidium bromide, FITC-Annexin-V/PI apoptosis assay kit, WST-1 assay kit and Hoechst 33342 were obtained from Beyotime Company of China. Antibody VEGFR2 (Flk-1 [C-1158], sc-504, rabbit polyclonal antibody raised against amino acids 1158–1345 of mouse Flk-1, Santa Cruz Biotechnology, USA). MMP-2 (H-76, sc-10736, rabbit monoclonal antibody against human MMP-2, Santa Cruz Biotechnology, USA). MMP-9 (H-129, sc-10737, rabbit monoclonal antibody against human MMP-9, Santa Cruz Biotechnology, USA). GAPDH (AG019, Primary antibodies used were mouse antibodies specific for the GAPDH, Beyotime Institute of Biotechnology, Jiangsu, China). F-actin (Mouse monoclonal [NH3] to F-actin, Abcam). β-actin (AA128, Primary antibodies used were mouse antibodies specific for the β-actin, Beyotime Institute of Biotechnology, Jiangsu, China). Secondary antibodies goat anti‑mouse (A0216, Beyotime Institute of Biotechnology, Jiangsu, China). Goat anti‑rabbit (A0208, 1:1000, Beyotime Institute of Biotechnology, Jiangsu, China).

### Synthesis and characterization of CQDs/Cu_2_O

Synthesis and characterization of CQDs/Cu_2_O have been discussed in our previous publications. [42, 43].

### Cell culture

All cell lines including human ovarian cancer cells (SKOV3), human cervical cancer cells (HeLa), human lung adenocarcinoma cells (A549), human colorectal cancer cells (HT-29, HCT116), normal mouse embryonic fibroblasts cells (BABL-3T3), normal human epithelial kidney cells HEK293T, normal mouse macrophage cells J774A1) were purchased from shanghai life science of chinese academy of sciences. The BABL-3T3 cells were grown in high glucose DMEM containing 10% FBS (Fetal bovine serum), while other cells were grown in low glucose DMEM containing 10% FBS. HUVECs were isolated from term umbilical cord veins using collagenase and cultured in DMEM supplemented with 20% FBS. All cell lines were grown at 37 °C in a humidified 5% CO_2_ atmosphere. HUVEC cells were used within 6 passages All cells were grown at 37 °C with 5% CO_2_ in a humidified incubator.

### MTT assay

The cytotoxicity of all cells was detected by MTT assay. 1 $\times$ 10^4^ cells were grown in 96-well plates for 24 h. Cells were incubated with 1.56, 3.12, 6.25, 12.5 and 25 µg mL^−1^ of CQDs/Cu_2_O for 24–72 h. Cells incubated PBS instead of CQDs/Cu_2_O were used as control. Then cells were treated with MTT (20 μL, 5 mg mL^−1^) for 4 h. 150 μL of DMSO was added after removing the medium. Microplate spectrophotometer (Spectra Max 190) was used to measure the absorbance at 490 nm. The assay was repeated 3 times and all experiments were carried out in duplicate. The inhibition rate (%) = (OD_control_ – OD _sample_)/OD_control_ $\times$ 100%. The half-maximal inhibitory concentration (IC_50_) was measured when the inhibition rate to half that of the control.

### WST-1 assay

1 $\times$ 10^4^ SKOV3 cells were grown in 96-well plates for 24 h. Cells were incubated with1.56, 3.12, 6.25, 12.5 and 25 µg mL^−1^ of CQDs/Cu_2_O for 24 h. 20 μL of WST-1 was added and incubated 1 h. The absorbance were measured by Microplate spectrophotometer (Spectra Max 190) at 450 nm. The assay was repeated 3 times and all experiments were carried out in duplicate. The inhibition rate (%) = (OD_control_ – OD _sample_)/ OD_control_ × 100%. The half-maximal inhibitory concentration (IC_50_) was measured when the inhibition rate to half that of the control.

### AO/EB staining

After treatment with 12.5 µg mL^−1^ CQDs/Cu_2_O, CQDs, or Cu_2_O for 24 h, SKOV3 cells were trypsinized and harvested. Cells in suspension were stained with 5 μg mL^−1^ AO/ EB for 10 min. Then cells were placed on a glass slide and analyzed using an Olympus IX73 fluorescent microscope at 545 nm.

### Hoechst 33342 staining

After treatment with 12.5 µg mL^−1^ CQDs/Cu_2_O, CQDs, or Cu_2_O for 24 h, SKOV3 cells were trypsinized and harvested. Cells were fixed for 10 min by 4% paraformaldehyde after washing 3 times using ice-cold PBS. SKOV3 cells were stained with Hoechst 33342 for 15 min after washing 3 times with PBS. Cells were analyzed using a Olympus IX73 fluorescent microscope at 350 nm.

### Flow cytometric analysis of cell cycle

After treatment with CQDs/Cu_2_O, CQDs, or Cu_2_O for 24 h, SKOV3 cells were trypsinized and harvested. Cells were fixed with 70% ethanol for 12 h at 4 °C, centrifuged (3000 rpm, 15 min) and washed using PBS, stained by PI for 20 min. Cells were subsequently analyzed by flow cytometer. The total number of cells was 10,000.

### Apoptosis detection by Annexin V staining

SKOV3 cells were treated with CQDs/Cu_2_O (3.12, 6.25, 12.5 µg mL^−1^). Cells were trypsinized and harvested, stained with FITC-Annexin-V and PI for 15 min in dark. Then tested by a flow cytometer. The number of cells was 10,000.

### Phalloidin staining

SKOV3 were treated with CQDs/Cu_2_O (3.12, 6.25, 12.5, 25 µg mL^−1^). Then cells were fixed using 4% paraformaldehyde, washed with PBS, and permeabilized by 0.1% Triton X-100 for 15 min. After washing by PBS, cells were then incubated with FITC-conjugated phalloidin. Cells were stained using PI for 10 min, then analyzed with Olympus IX73.

### Wound healing assay

SKOV3 were seeded onto six-well plates. When cells grown to 90% confluence to form monolayers, cells were scratched by a pipette tip. Subsequently, CQDs/Cu_2_O (6.25, 12.5, 25 µg mL^−1^) were added and incubated with SKOV3 cells for 0, 6, 12 and 24 h. The number of cells migrated into the scratch area was quantified under fluorescent microscope. The migration inhibition rate of a sample was calculated by the following equation: inhibition percentage = (N_Control_ − N_Sample_)/ N_Control_ × 100%, where N_Control_ is the average total number of migrated cells in three groups of control, N_Sample_ is the average total number of migrated cells in three groups of CQDs/Cu_2_O.

### Tube structure formation assay

HUVEC cells (2 $\times$ 10^4^ cells per well), VEGF (10 ng mL^−1^) and different concentration of CQDs/Cu_2_O were added into 96-well plates which coated by Matrigel for 12 h. Tubular network were observed and quantified by the measuring tool of the software in randomly chosen five fields under a fluorescence microscope (Olympus IX73, Japan). The inhibition (%) = (length_Control_ − length _Sample_)/length _Control_ $\times$ 100%.

### Western blot

SKOV3 cells treated by CQDs/Cu_2_O (6.25, 12.5, 25 µg mL^−1^) for 24 h. After harvested and washed by cold PBS, cells were incubated with cell lysate for 1 h at 4 °C on ice. The proteins were extracted by centrifuging for 15 min (14,000 rpm, 4 °C). 10% SDS-PAGE was used to separate the extracted proteins. After proteins transferred, membranes were treated with blocking agents. The membranes were incubated with primary antibodies (VEGFR2 1:200, GAPDH 1:3000, MMP-2 1:200, MMP-9 1:200, F-actin 1:200, β-actin 1:1000, GAPDH 1:1000) at 4˚C overnight. After washed 3 times, the membranes were incubated with secondary antibodies goat anti‑mouse or goat anti‑rabbit at room temperature for 1 h. Washing 3 times with PBST, the membranes were treated using chemiluminescence agents.

### RNA sequencing

SKOV3 cells were treated with CQDs/Cu_2_O (3.12,12.5 μg mL^−1^) for 24 h. Total RNA of cells treated by CQDs/Cu_2_O was extracted by TRIzol reagent. Sequencing libraries were tested by the Illumina HiSeq xten/NovaSeq 4000 sequencer. A fold change of 2 were used as the threshold. *P* < 0.05 was indicated statistically significant.

### Statistical analysis

All values represent mean ± SD, n = 3. The significant levels were assessed by Student’s t-test. *P* < 0.05 was indicated the statistically significant.

## Results and discussion

### MTT assays

**Fig. 1:**
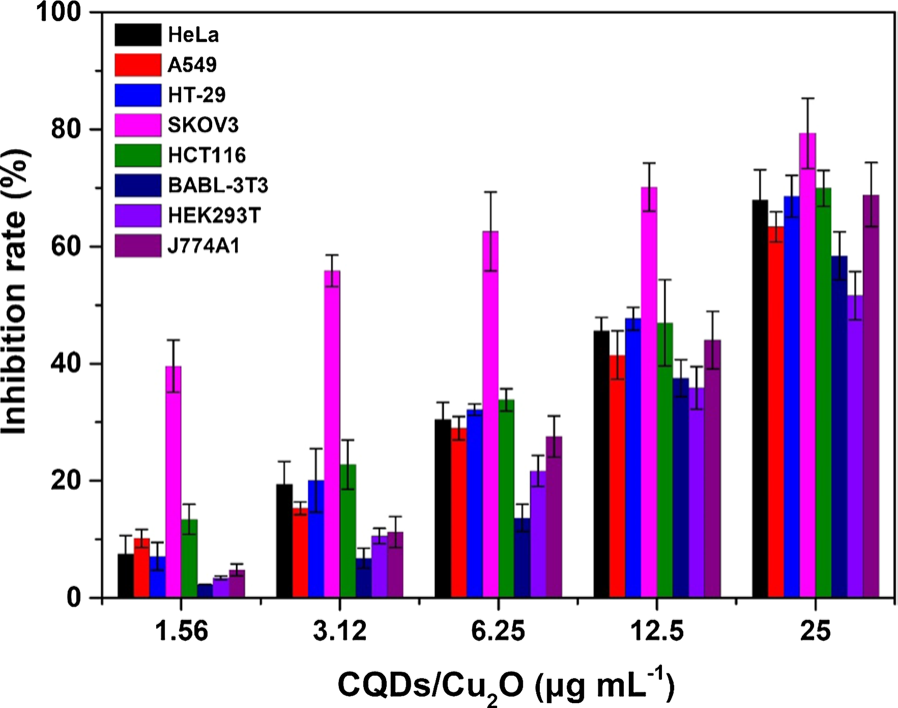
Differential cytotoxicity of CQDs/Cu_2_O in cancer cells (HeLa, A549, HT-29, SKOV3, HCT116) and normal cells (BABL-3T3, HEK293T, J774A1) by the MTT assay for 24 h

The effect of CQDs/Cu_2_O, Cu_2_O on cell cytotoxicity was tested in cancer cells (HeLa, A549, HT-29, SKOV3, HCT116), and normal cells (BABL-3T3, HEK293T, J774A1) by the MTT assay. Cells were treated with CQDs/Cu_2_O or Cu_2_O (1.56, 3.12, 6.25, 12.5, 25 µg mL^−1^) for 24–72 h. The effect of CQDs was detected in HeLa, SKOV3 and BABL-3T3 cells by MTT assay. The results were shown CQDs were non-toxic and biocompatibility, which is agreed with previous findings [34]. As shown in Fig. 1, CQDs/Cu_2_O displayed cytotoxicity in a concentration-dependent manner in all tested cells, and SKOV3 cells displayed the highest inhibitory rate for proliferation among all cells. To further verify the results of MTT assay, a second mitochondrial activity-based assay, WST-1, was employed to measure the cytotoxicity of CQDs/ Cu_2_O in SKOV3 cells. WST data showed that CQDs/Cu_2_O inhibited the growth of SKOV3 cells in a dose-dependent manner, the value of IC_50_ obtained by WST assay (IC_50_ = 1.46 µg mL^−1^) was consistent with the result from MTT assay (IC_50_ = 1.50 µg mL^−1^). It was suggested the MTT assay can be used to evaluate the cytotoxicity of CQDs/Cu_2_O.

Interestingly, when the concentration of CQDs/Cu_2_O was lower than 12.5 µg mL^−1^, a discriminative difference was identified for the cytotoxicity between SKOV3 cancer cells and other cancer cells, even normal BABL-3T3, HEK293T and J774A1 cells. This is important because developing an anticancer drug that can effectively distinguish between tumor and normal cells is currently the greatest challenge [21].

In general, the CQDs/Cu_2_O composite displayed cytotoxic activity against cancer and normal cells in the IC_50_ (half maximal inhibitory concentration) range of 0.85 to 22.4 µg mL^−1^ (Table 1). The IC_50_ value of CQDs/Cu_2_O against cells was lower than Cu_2_O and CQDs were non-toxic. It was indicated that the cytotoxicity of the CQDs/Cu_2_O composite was higher than that of the CQDs and Cu_2_O. In addition, the leakage of copper into the medium was undetectable at 37 °C after 72 h by ICP-AES (coupled plasma-atomic emission spectrometry) measurement, suggesting CQDs/Cu_2_O displayed high stability and the CQDs/Cu_2_O plays a leading roles in cytotoxic activity. Moreover, the IC_50_ value of CQDs/Cu_2_O against SKOV3 cells was lower than above mentioned cancer cells and normal cells, which was approximately threefold lower than other tested cancer cells and approximately 12-fold lower than normal cells. It was further suggested CQDs/Cu_2_O was sensitive to SKOV3 cells.

**Table 1:** Comparison of the IC_50_ (μg mL^−1^) of CQDs/Cu_2_O composite and Cu_2_O in different cell lines

Cells	IC_50_ (CQDs/Cu_2_O)			IC_50_ (Cu_2_O)		
	24 h	48 h	72 h	24 h	48 h	72 h
HeLa	10.6	9.5	4.9	27.8	23.9	28.1
A549	15.8	12.3	10.5	31.3	24.7	23.6
HT-29	7.1	4.0	2.5	16.2	14.3	15.5
SKOV3	1.5	0.9	0.85	15.7	13.9	12.2
HCT116	9.4	6.9	5.8	15.0	11.7	14.8
BABL-3T3	18.9	17.8	16.9	29.5	27.4	18.3
HEK293T	22.4	16.3	11.7	24.8	19.6	15.9
J774A1	20.1	16.4	14.2	23.8	18.9	16.6

On the other hand, compared with commercial anticancer agents such as ART and OXA at different times, the cytotoxicity of CQDs/Cu_2_O was better than that of ART and OXA against cancer cells (Table 2). The IC_50_ of CQDs/Cu_2_O in SKOV3 cells (IC_50_ = 0.85 µg mL ^−1^) was approximately 75-fold lower than that of OXA (IC_50_ = 64.6 µg mL ^−1^) [21] and 114-fold lower than that of ART (IC_50_ = 96.9 µg mL ^−1^) [21] at 72 h. Therefore, CQDs/Cu_2_O may have a potential utility for effectively distinguishing between SKOV3 cells and other tested cells.

**Table 2:** Comparison of the IC_50_ (μg mL^−1^) of CQDs/Cu_2_O, OXA and ART in different cell lines

Cells	IC50 (CQDs/Cu_2_O)			IC50 (OXA)			IC50 (ART)		
	24 h	48 h	72 h	24 h	48 h	72 h	24 h	48 h	72 h
HeLa	10.6	9.5	4.9	84.8 [21]	28.5 [21]	20.0 [21]	126.5 [21]	62.7 [21]	43.6 [21]
A549	15.8	12.3	10.5	219.8 [21]	128.3 [21]	27.0 [21]	160.7 [21]	66.0 [21]	31.1 [21]
HT-29	7.1	4.0	2.5	47.81	32.65	26.82	63.41	50.24	43.60
SKOV3	1.5	0.9	0.85	241.5 [21]	120.8 [21]	64.6 [21]	280.8 [21]	121.1 [21]	96.9 [21]
HCT116	9.4	6.9	5.8	62.9	41.2	28.6	40.3	22.6	21.4
BABL-3T3	18.9	17.8	16.9	77.8 [21]	34.2 [21]	13.8 [21]	118.2 [21]	52.4 [21]	36.6 [21]

### RNA‑Seq data and identification of differentially expressed genes (DEGs)

**Fig. 2:**
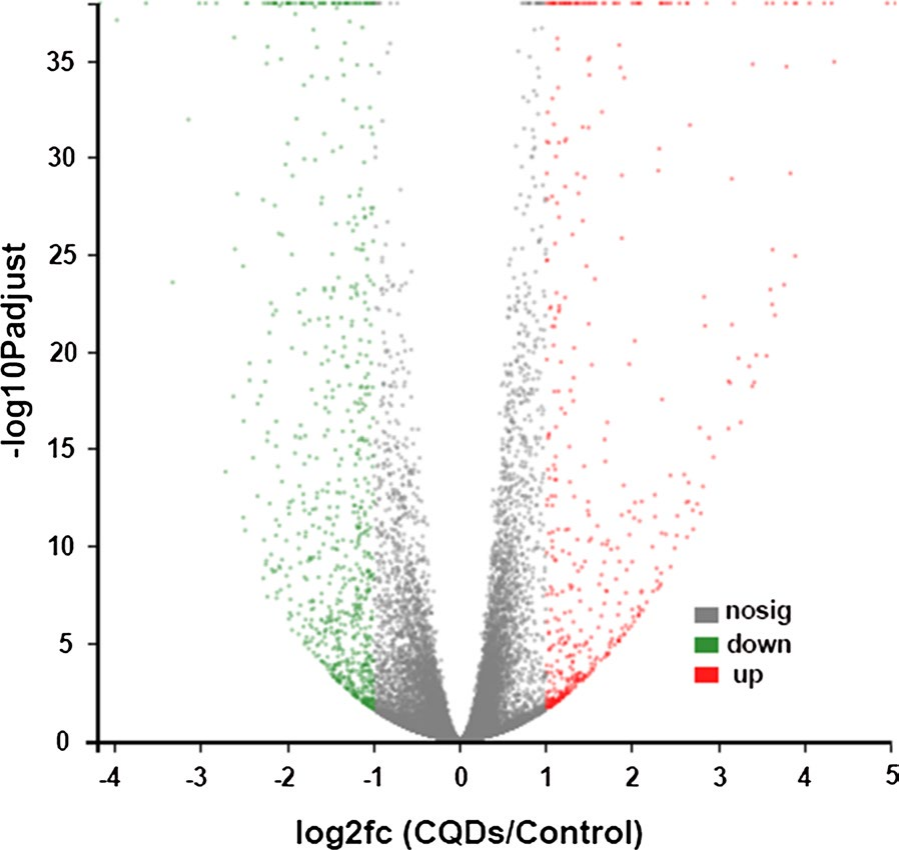
Volcano plot of DEGs in SKOV3 cells after treated with CQDs/Cu_2_O. The red dots expressed the upregulated genes and green dots expressed the downregulated genes. The black dots indicate the genes with no significant differential expression

Six sequencing libraries including CQDs/Cu_2_O treatment libraries (CQDs1, CQDs2, CQDs3) and control group libraries (Control1, Control2, Control3) were successfully constructed and subsequently sequenced (Table 3). The RNA-Seq of six samples produced 37.95 million raw reads and 37.05 million high quality clean reads. It was shown that 95.47–97.77% of the reads were successfully mapped to the Homo genome (Table 3). Compared to the control, fragments per kilobase of transcript per million fragments (FPKM) analysis indicated that a total of 1251 transcripts at a fold change > 2 and false discovery rate (FDR) < 0.05 were differentially expressed, among which, 495 genes were upregulated while 756 were downregulated (Fig. 2).

**Table 3:** The raw data from RNA-Seq analysis of the CQDs/Cu_2_O and control group

Sample	Raw reads	Clean reads	Total mapped	Q30 (%)	GC content (%)
Control1	56,611,380	54,934,068	52,445,514(95.47%)	93.71	50.23
Control2	68,457,650	66,554,960	63,613,738(95.58%)	93.97	50.25
Control3	60,574,334	59,402,794	58,070,632(97.76%)	96.09	50.49
CQDs1	63,142,992	61,528,818	58,951,428(95.81%)	94.14	50.40
CQDs2	65,707,806	63,946,010	61,244,702(95.78%)	93.97	50.23
CQDs3	65,006,850	63,690,950	62,268,500(97.77%)	96.08	50.88

In order to determine the differentially expressed genes at lower doses and elucidate the mechanism of selective inhibition of SKOV3 cells by CQDs/Cu_2_O, the RNA sequencing analysis were performed after SKOV3 cells treated with 3.12 μg mL^−1^ CQDs/Cu_2_O for 24 h. Compared to the control, fragments per kilobase of transcript per million fragments (FPKM) analysis indicated that a total of 1011 transcripts at a fold change > 2 and false discovery rate (FDR) < 0.05 were differentially expressed, among which, 381 genes were upregulated while 630 were downregulated. There was no significant difference between the high concentration group and the low concentration group. On the one hand, the enriched GO terms in the DEGs of cells treated by 3.12 μg mL^−1^ CQDs/Cu_2_O have also been provided in supporting information (Additional file 1: Figure S1). As shown in Additional file 1: Figure S1, the distribution of enriched GO terms of cells treated by 3.12 μg mL^−1^ CQDs/Cu_2_O was similar with that treated by 12.5 μg mL^−1^ CQDs/Cu_2_O. Moreover, DEGs analysis results showed that the angiogenesis-related genes (Maspin and TSP1) were significantly upregulated after 3.12 μg mL^−1^ CQDs/Cu_2_O treatment, which was also consistent with those of higher doses treatment.

### GO and KEGG analysis of DEGs

**Fig. 3:**
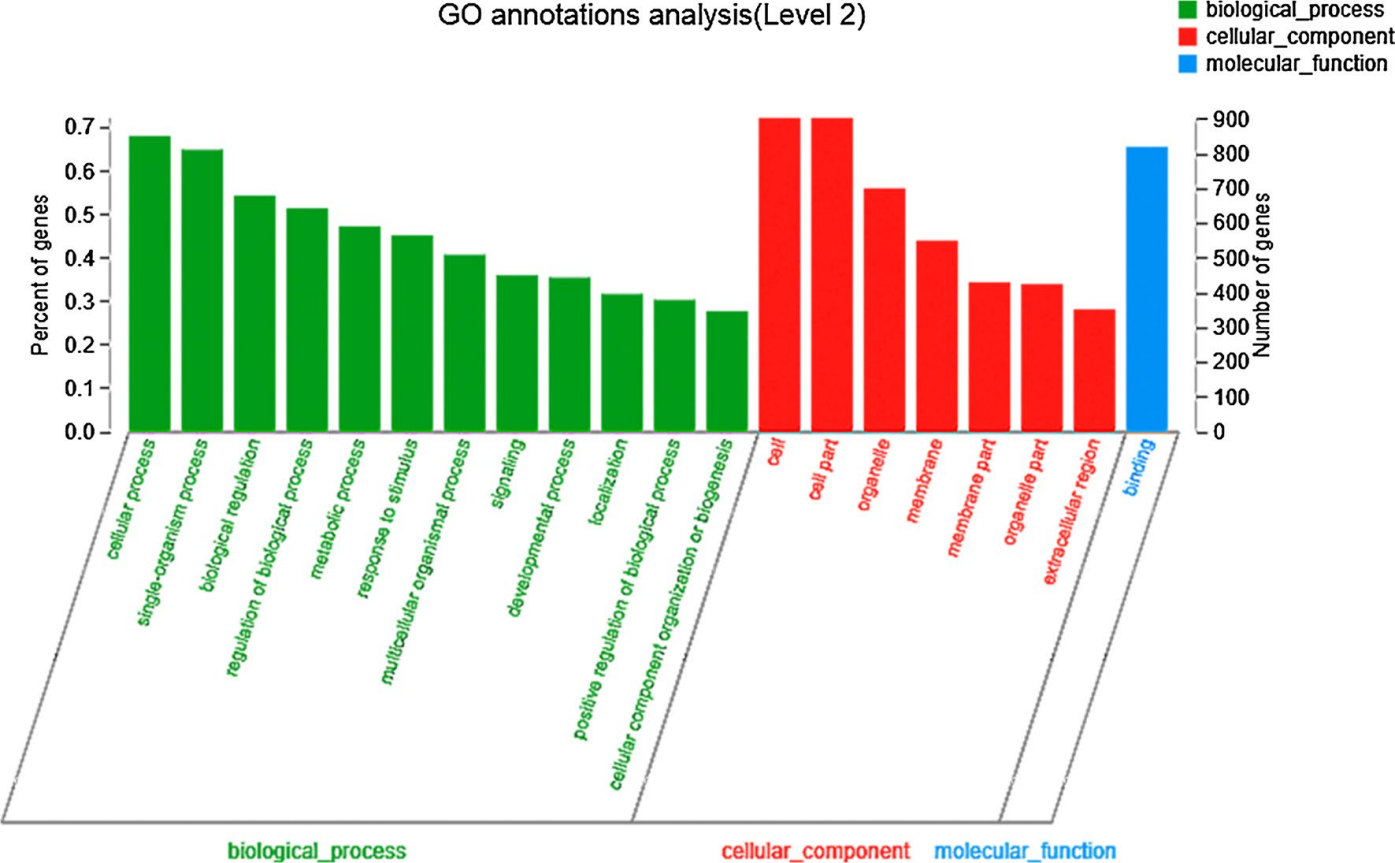
The enriched GO terms in the DEGs of cells treated by 12.5 μg mL^−1^ CQDs/Cu_2_O. The green, red, and blue bars represent the terms of biological process, cellular component, and molecular function, respectively

Gene Ontology (GO) was used to analyze the obtained DEGs, where three main ontologies such as biological process, cellular component, and molecular function performed. As shown in Fig. 3, the top 20 most enriched GO terms are summarized. In the category of biological process, the most abundant groups were “cellular process”, “single-organism process”, and “biological regulation”. Within the cellular component category, the “cell”, “cell part”, and “organelle” were identified as the most enriched GO terms. The molecular functional groups of DEGs were also related to “binding”.

GO enrichment analysis was also used to identify significantly enriched BP, CC, or MF terms in DEGs during CQDs/Cu_2_O treatment. As shown in Table 4, there were 10, 8, and 6 ontology terms enriched in BP, CC, and MF, respectively. The enriched BP terms mainly included the regulation of the apoptotic process, programmed cell death, and cell motility, indicating that CQDs/Cu_2_O plays a key role in the induction of SKOV3 cell apoptosis and the regulation of cell movement. The enriched CC terms mainly included proteinaceous extracellular matrix and integral components of the plasma membrane, suggesting that CQDs/Cu_2_O affected the extracellular matrix and plasma membrane. The enriched MF terms included calcium ion binding, nucleic acid binding, receptor binding, etc.

**Table 4:** GO enrichment analysis of the DEGs during CQDs/Cu_2_O treatment (*P* value < 0.01)

Description	GO ID	Term Type	Number	*P*-value uncorrected	*P*-value corrected
Regulation of apoptotic process	GO:0042981	BP	138	1.89891E-10	0
Regulation of programmed cell death	GO:0043067		138	2.3484E-10	0
Regulation of locomotion	GO:0040012		91	2.44938E-10	0
Regulation of cell death	GO:0010941		150	2.78134E-10	0
Regulation of localization	GO:0032879		216	2.82001E-10	0
Response to organic substance	GO:010033		168	3.03004E-10	0
Positive regulation of response to stimulus	GO:0048584		184	3.05792E-10	0
Regulation of cell motility	GO:2000145		84	3.254E-10	0
Positive regulation of signal transduction	GO:0009967		134	3.27693E-10	0
Anatomical structure development	GO:0048856		293	1.89891E-10	0
Proteinaceous extracellular matrix	GO:0005578	CC	57	5.76E-11	0
Extracellular matrix	GO:0031012		70	1.49E-10	0
Integral component of plasma membrane	GO:0005887		129	1.9E-10	0
Plasma membrane part	GO:0044459		227	2.99E-10	0
Intrinsic component of plasma membrane	GO:0031226		136	3.4E-10	0
Extracellular space	GO:0005615		153	3.46E-10	0
Extracellular region	GO:0005576		219	3.7E-10	0
Extracellular region part	GO:0044421		321	3.82E-10	0
Calcium ion binding	GO:0,005,509	MF	79	6.87E-10	0
Nucleic acid binding	GO:0003676		145	1.02E-09	0
Heparin binding	GO:0008201		26	1.12E-07	0
Receptor binding	GO:0005102		127	1.67E-07	0
Sulfur compound binding	GO:1901681		32	2.52E-07	0
Glycosaminoglycan binding	GO:0005539		29	9.63E-07	0.002

**Fig. 4:**
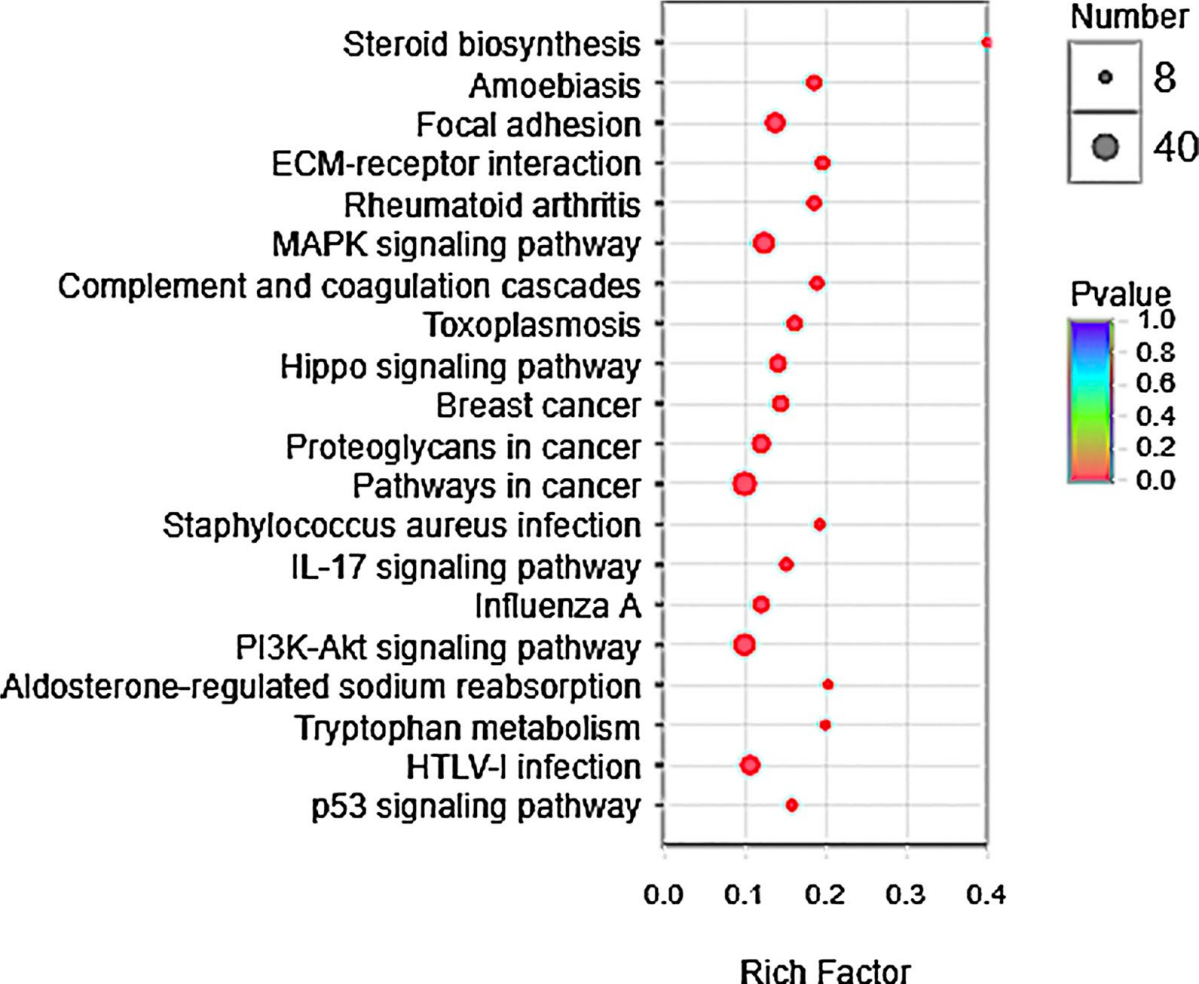
A bubble diagram of the significant enrichment pathways through KEGG analysis of DEGs (*P* < 0.05). The size and color of bubble indicated the number of gene enriched in certain pathway and the *P*-value, respectively. The rich factor = (the number of DEGs mapped to a certain pathway)/ (the total number of genes mapped to this pathway)

To determine the effect of CQDs/Cu_2_O on the functions of DEGs, the KEGG data base was used to analyze annotated pathways of DEGs. The result indicated that DEGs were significantly enriched in 20 pathways after treated with CQDs/Cu_2_O in Fig. 4 (*P* corrected < 0.05). According to the number of DEGs, the three pathways such as steroid biosynthesis, focal adhesion, and ECM-receptor interaction were the most enriched pathways. Based on the lowest *P* value, steroid biosynthesis was among the most notable enriched pathways.

### CQDs/Cu_2_O induced apoptosis of SKOV3 cells

**Fig. 5:**
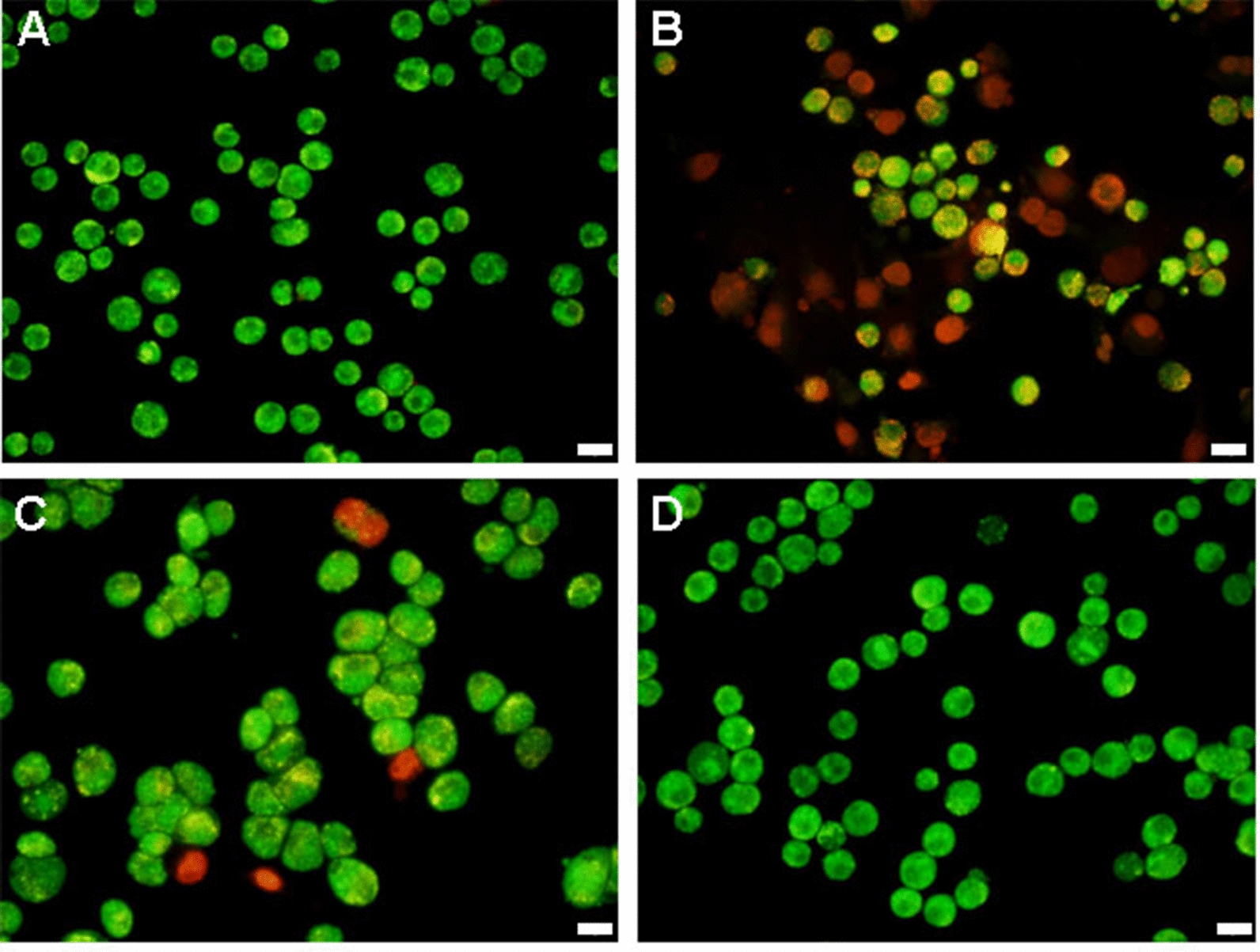
Flu or escence photomicrographs changes by AO/EB staining of SKOV3 cells after treatment with CQDs/Cu_2_O (**B**), CQDs (**C**), or Cu_2_O (**D**) for 24 h compared to controls (**A**), respectively. Scale bar 20 μm.

Transcriptome analysis revealed that apoptotic process and programmed cell death were significantly affected. AO/EB (acridine orange/ethidium bromide), Hoechst 33342, and FITC-Annexin-V/PI were used to demonstrate apoptosis. AO is absorbed by viable and nonviable cells to emit green fluorescence, while EB is absorbed by nonviable cells to emit red fluorescence. Therefore, viable cells displayed uniform bright green fluorescence with organized structure. The apoptotic cells showed red to orange fluorescence with fragmented chromatin, and the necrotic cells showed red fluorescence with swollen [44]. As shown in Fig. 5, cells appeared healthy with a green nucleus in the control group. Some of the cells incubated with the CQDs/Cu_2_O samples had an orange nuclei and fragmented chromatin, indicating that they were either in necrosis or at the late stage of apoptosis. In addition, only few necrotic cells with uniformly red nuclei and an organized structure appeared after treatment with the same concentration of Cu_2_O. However, there was no significant change in the CQD group. These results indicate that CQDs/Cu_2_O induces necrosis or apoptosis of SKOV3 cells, and the effect of the CQDs/Cu_2_O composite is better than that of CQDs and Cu_2_O.

**Fig. 6:**
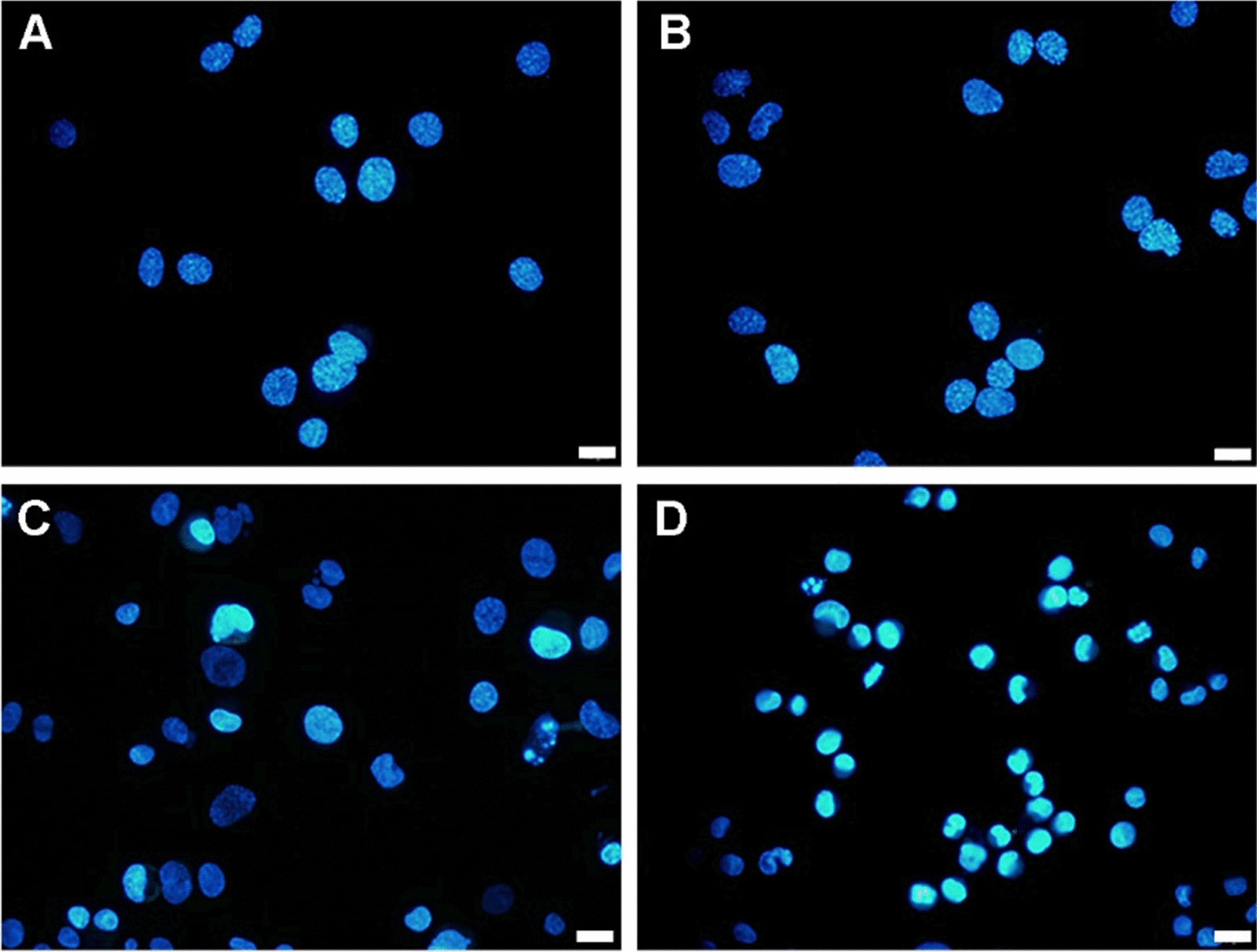
Fluorescence photomicrographs changes by Hoechst 33342 staining of SKOV3 cells after treatment with CQDs (**B**), Cu_2_O (**C**) and CQDs/Cu_2_O (**D**) for 24 h compared to controls (**A**), respectively. Scale bar 20 μm

The apoptotic effects of CQDs/Cu_2_O on SKOV3 cells were determined by staining with Hoechst 33342. Nuclear condensation & DNA fragmentation are considered to be typical features of apoptosis. SKOV3 cells were treated with 12.5 μg mL^−1^ CQDs/Cu_2_O for 24 h. As shown in Fig. 6, cells displayed a uniformly blue fluorescence in the control group and CQDs group. CQDs/Cu_2_O treated cells showed nuclear shrinkage, cytoplasmic blebbing, and chromatin condensation, including bright blue dots in the nuclei, thereby representing nuclear fragmentation, whereas a small number of cells were chromatin condensation in Cu_2_O group. These observations further indicated CQDs/Cu_2_O can induce SKOV3 cells apoptosis, which is better than both CQDs and Cu_2_O.

**Fig. 7:**
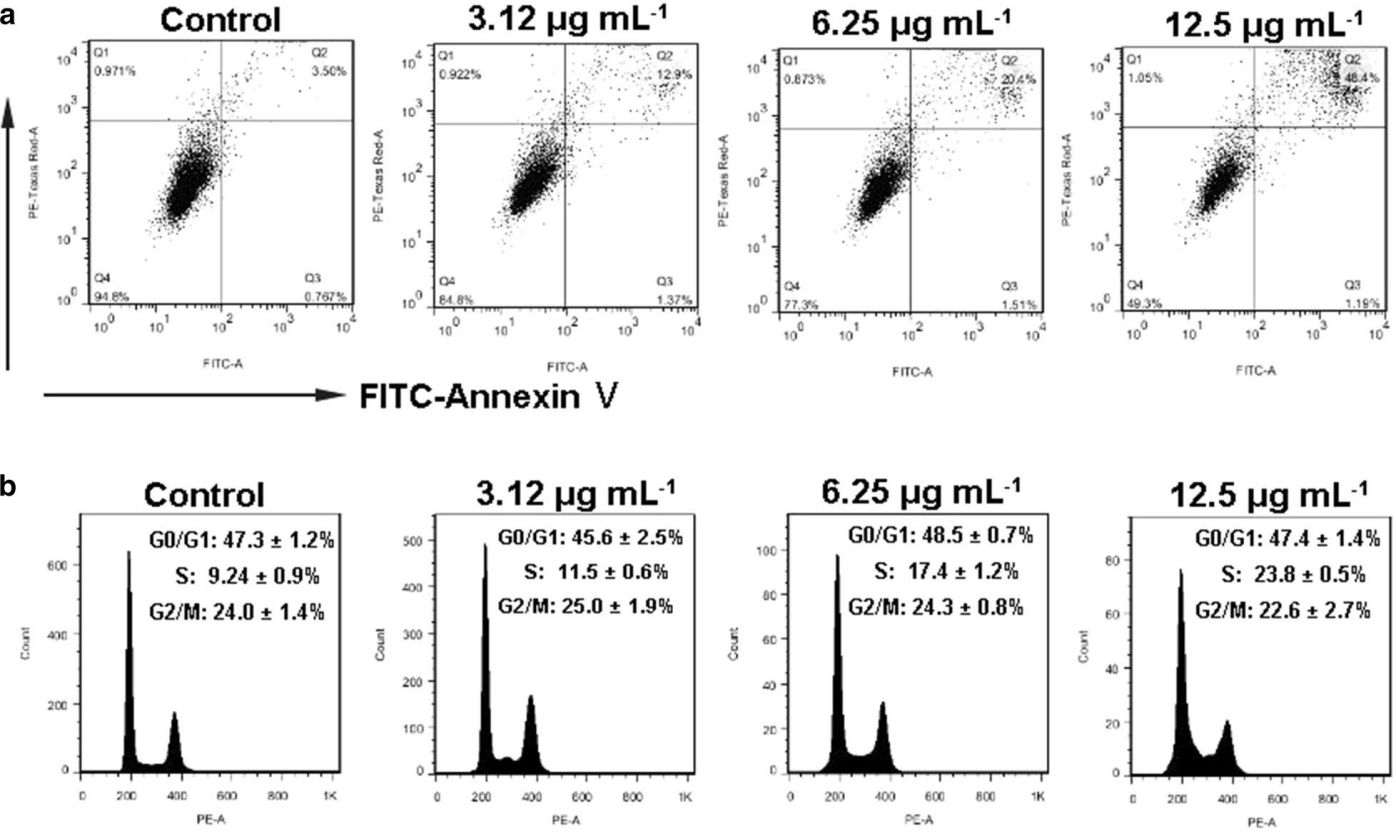
Effects of CQDs/Cu_2_O on the apoptosis and cell cycle distribution by flow cytometry. **a** Flow cytometric analysis of SKOV3 cells incubated with CQDs/Cu_2_O for 24 h using an apoptosis kit with dual fluorescence of annexin V-FITC/PI. **b** SKOV3 were treated with CQDs/Cu_2_O for 24 h and stained using PI to determine the content of DNA. The experiments were tested at least 3 times

A flow cytometric analysis via FITC-Annexin-V/PI assay was used to further confirm the CQDs/Cu_2_O-induced apoptosis. As shown in Fig. 7a, as the concentration of CQDs/Cu_2_O increased from 3.12 ~ 12.5 μg mL^−1^, the proportion of cells in the upper right quadrants increased from 3.5% to 48.4%, however, the proportions of other quadrant cells did not change significantly. This data also confirm that CQDs/Cu_2_O can effectively induce SKOV3 cell apoptosis.

### Determination of the cell cycle

The cell cycle, which includes DNA replication, mitosis, and cytokinesis, is a major event in cell division. It has been proven that deregulation of the cell cycle is related to numerous carcinogenic processes [45]. Nanomaterials, such as Se, Ag, ZnO, Ag-ZnO, TiO_2_ NPs, have been reported to arrest cell cycle to suppress cancer cell proliferation [46–49]. Cu-based nanomaterials also play a critical role in regulating the cell cycle. For example, cuprous oxide nanoparticles (CONPs) suppressed the proliferation of HeLa cells and caused cell cycle arrest in G0/G1 [36]. A previous study reported that Cu-MOF (HKUST-1) has the potential to inhibit the proliferation of SKOV3 by cell cycle arrest in G2/M. [30] To determine whether apoptosis is involved in the cell cycle effect of CQDs/Cu_2_O, SKOV3 cells were treated by CQDs/Cu_2_O and subjected to flow cytometry analysis (Fig. 7b). As shown in Fig. 7b, CQDs/Cu_2_O led to accumulation of cells in the S phase (9.24 ± 0.9% in control group versus 23.8 ± 0.5% in 12.5 μg mL^−1^ CQDs/Cu_2_O treated group (*P* < 0.001), while there was no significant change in the G0/G1 and G2/M phase. The results showed that the SKOV3 cells are arrested at S phase after treatment by CQDs/Cu_2_O, indicating that mitosis and proliferation of cancer cells were inhibited.

### Effects of CQDs/Cu_2_O on the SKOV3 migration

**Fig. 8:**
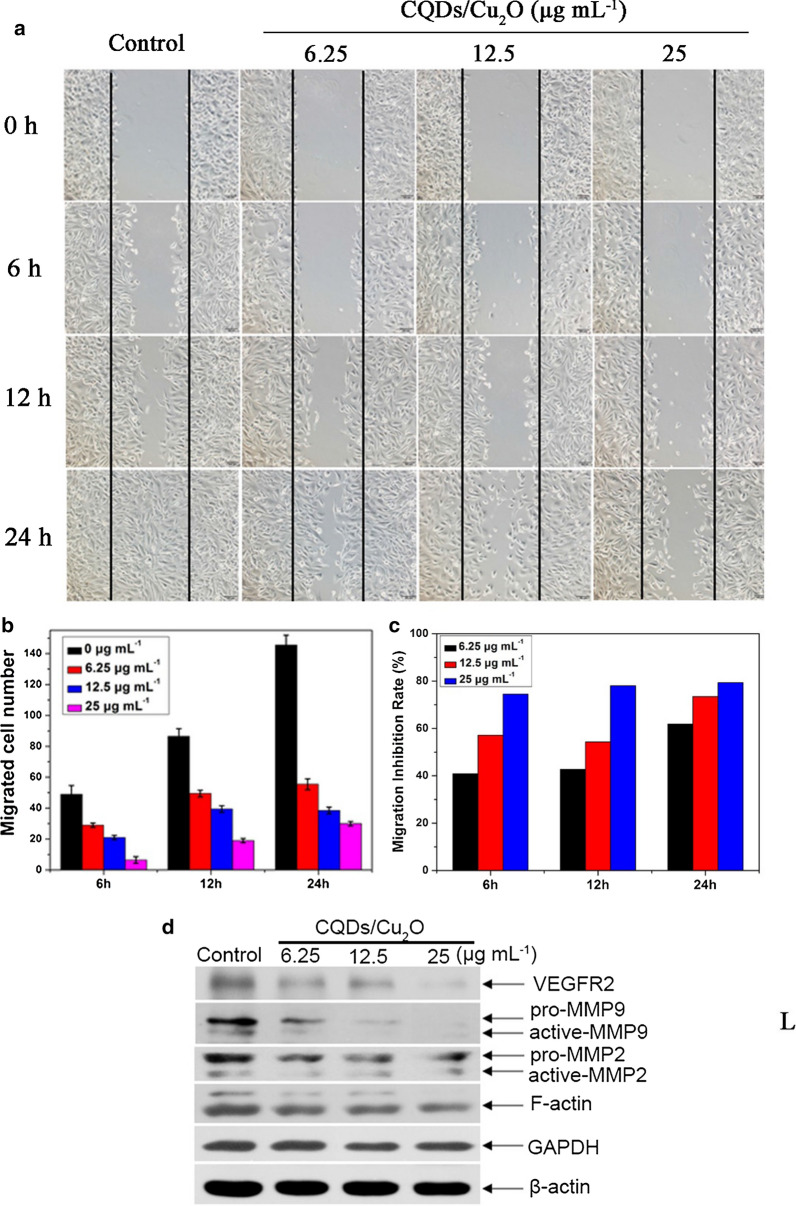
Effect of CQDs/Cu_2_O on SKOV3 cell migration by the wound-healing assay. **a** The “scratch” was produced by scraping the monolayer SKOV3 cells using a pipette tip after grown to form a confluent monolayer. (Scale bar 100 μm). The relative migration activity (**b**) and the inhibition rate of migration (**c**). The experiments were tested at least 3 times

Ovarian cancer is the one of the leading causes of death in gynecological malignancies. Local recurrence and metastasis are considered to be the main causes of treatment failure for ovarian cancer. It was critical to suppress the metastasis of cancer cells for the development of effective drugs and therapies. GO enrichment analysis revealed that CQDs/Cu_2_O regulated cell motility. Hence, the wound-healing assay was used to detect the amount of migrated cells and the width of scratch after treatment with CQDs/Cu_2_O. As shown in Fig. 8a, the width of scratch increases with increasing concentrations of CQDs/Cu_2_O and prolonging reaction time. As well as the amount of migrated cells decreased after treated with CQDs/Cu_2_O (Fig. 8b). The migration inhibition rate increased from 61.8 to 79.4% after incubation with CQDs/Cu_2_O (Fig. 8c). When cells treated with 25 μg mL^−1^ CQDs/Cu_2_O, the migration inhibition rate doesn't show significant change at 6, 12 and 24 h, mainly due to the strong cytotoxicity of high concentration of CQDS /Cu_2_O, and the inhibitory effect on cell migration was shown in a short time and maintained for a period of time. CQDs/Cu_2_O was demonstrated to exhibit remarkable concentration- and time-dependency, suggesting that it has the potential to suppress SKOV3 cell migration.

We found that the expression of MMP-2/9 sharply decreased after treatment with CQDs/Cu_2_O for 24 h by western blot analysis (Fig. 8d). It has been reported the decrease of MMPs expression is considered to be the main factor for the inhibition potential of migration and angiogenesis. Carbon nanocapsules (HMCNs) and fullerene-based nanoparticle Gd@C_82_(OH)_22_ serve as potent migration inhibitors by downregulating MMP-2/9 [20, 50]. The results further indicated that CQDs/Cu_2_O effectively suppressed SKOV3 cell migration by regulating the expression of metalloproteinase.

### Effects of CQDs/Cu_2_O on HUVEC blood vessel formation

**Fig. 9:**
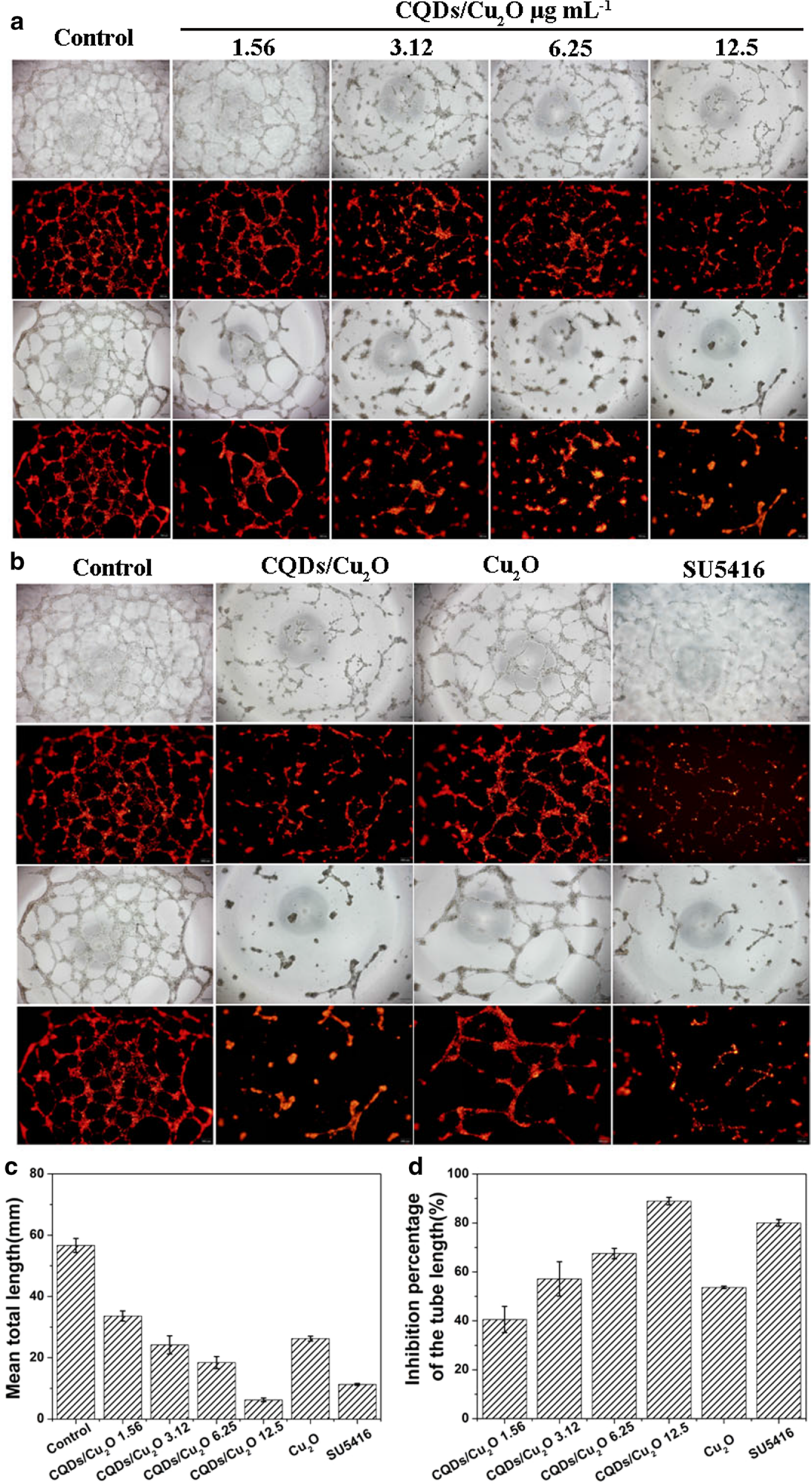
Dil-labeled HUVECs were inoculated in Matrigel-coated wells and induced blood vessel formation. Scale bar, 200 μm. **a** CQDs/Cu_2_O composite inhibited blood vessel formation. Scale bar, 200 μm. **b** The effect of 12.5 μg mL^−1^ CQDs/Cu_2_O (87 μM), Cu_2_O (87 μM) and SU5416 (87 μM) on blood vessel formation. **c** Cumulative tube length in four fields/well and the inhibition percentage of the tube length

Angiogenesis is required for tumor progression and metastasis to provide oxygen and nutrients. Targeting angiogenesis has become a unique perspective and strategy in anticancer therapy. DEGs analysis results showed that the SERPINB5 (known as Maspin) and THBS1 (known as TSP1) genes were significantly upregulated after CQDs/Cu_2_O treatment. To confirm whether CQDs/Cu_2_O has an anti-angiogenesis effect, a blood vessel formation in vitro model system was selected and the effect of CQDs/Cu_2_O on new blood vessel formation in HUVECs cells was assessed. VEGF was used to induce endothelial cell proliferation, migration, and differentiation into blood vessel structures. As shown in Fig. 9a a robust and complete blood vessel produced after HUVECs cells treated with VEGF within 12 h. CQDs/Cu_2_O effectively inhibited the length of blood vessel. A reduction of approximately 40 ~ 70% in total blood vessel length per field following treatment with 3.12 to 12.5 μg mL^−1^ CQDs/Cu_2_O for 6 h. When the time was extended to 12 h, the inhibition percentage increased from 60 to 90%, suggesting a dose-dependent and a time-dependent decrease. The viability of HUVEC cells was also determined by MTT assay. It was shown that the inhibition rate was lower than 20% when HUVEC cells were treated with CQDs/Cu_2_O for 12 h (Additional file 1: Figure S2), indicating that this concentration of CQDs/Cu_2_O was non-toxic over a short period and antiangiogenic activity was not caused by the cytotoxicity of CQDs/Cu_2_O.

Moreover, semaxanib (SU5416, 87 μM), as a selective inhibitor of VEGF, was used as the experimental control. CQDs/Cu_2_O also had better antiangiogenic activity than SU5416 and Cu_2_O (Fig. 9b). In Fig. 9c, the length of blood vessel induced by CQDs/Cu_2_O (12.5 μg mL^−1^) was about 1.8-fold shorter than the length of blood vessel induced by SU5416. The length was approximately 4.2-fold shorter than that caused by Cu_2_O. CQDs/Cu_2_O inhibited tube formation by ~ 90%, which was more than that with Cu_2_O (53%) and SU5416 (80%) (Fig. 9d). It has been reported 80 μg mL^−1^ AgNPs [19] suppressed about 80% blood vessel formation of bovine retinal endothelial cells. 12.5 μg mL^−1^ Fe-MIL-101 [21] effectively suppressed about 60% blood vessel formation of HUVEC cells. It was suggested that CQDs/Cu_2_O possessed better anti-angiogenic activity than AgNPs and Fe-MIL-101.

A variety of growth factors and cytokines are involved in regulating angiogenesis. VEGF, as one of the most important pro-angiogenic factors, combined to receptors VEGFR1 and VEGFR2, stimulating endothelial cell migration and blood vessel formation. [13, 17, 51] As shown in Fig. 8d, the expression of VEGFR2 decreased after treatment with CQDs/Cu_2_O. Altogether, our results indicated that angiogenesis mediated by VEGFR2 and MMP-2/9, indicating that CQDs/Cu_2_O may be a potential candidate in anti-angiogenic therapy.

### CQDs/Cu_2_O induced alterations in the cytoskeleton of SKOV3 cells

**Fig. 10:**
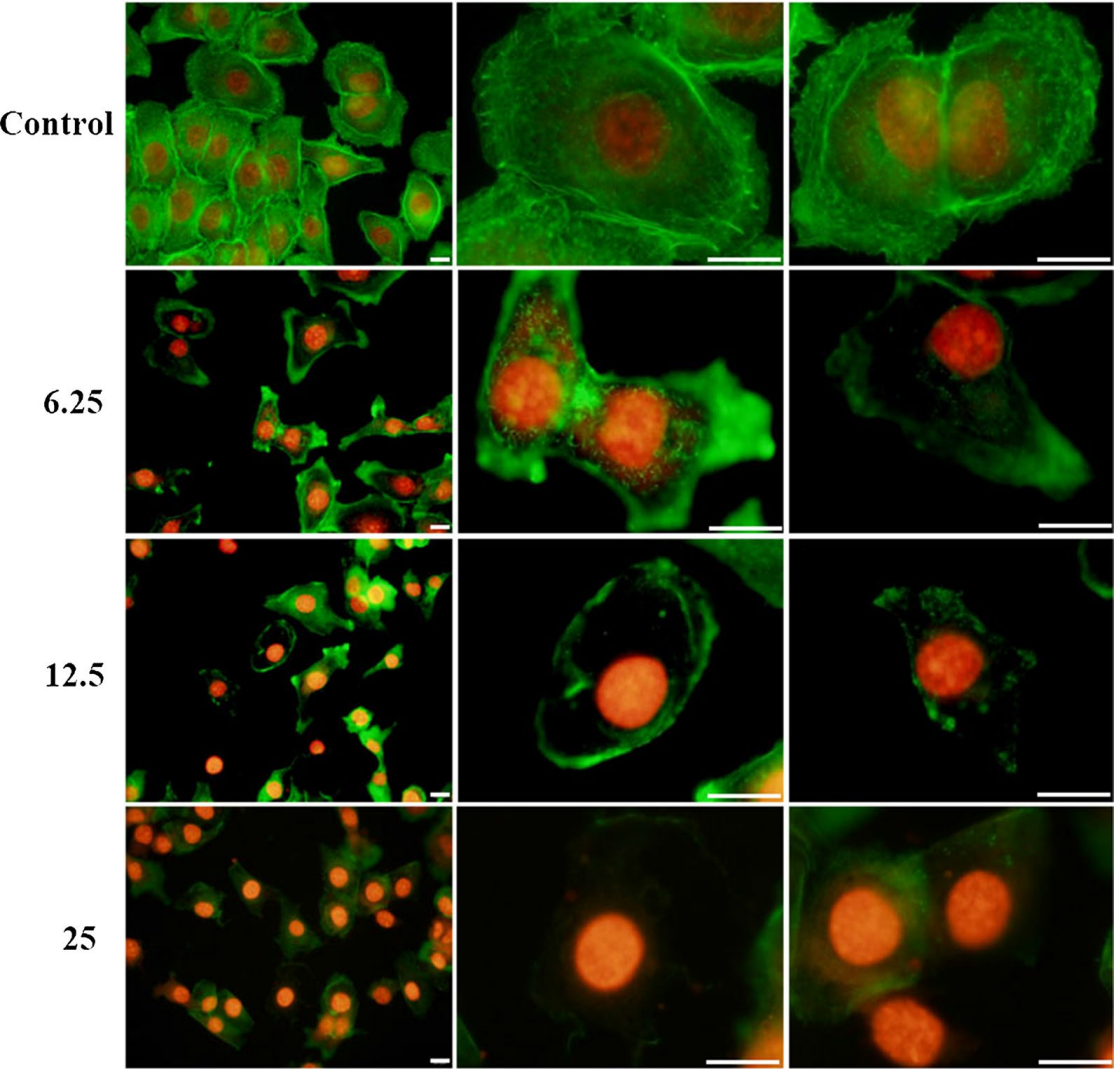
Effects of CQDs/Cu_2_O-stimulated disruption of the cytoskeleton. SKOV3 cells were stained using FITC-conjugated phalloidin and PI after treatment with CQDs/Cu_2_O (0, 6.25, 12.5, 25 μg mL^−1^) for 24 h. Images are representative of at least three experiments, Scale bar, 20 μm

The cytoskeleton, which has a variety of biopolymer networks such as F-actin, microtubules, and intermediate filaments, plays a fundamental role in all eukaryotic cells [52]. Dynamic regulation of the F-actin cytoskeleton is essential for many physiological cellular processes, including cell adhesion, migration, division, and apoptosis of cancer cells. We investigated the effect of CQDs/Cu_2_O on the F-actin cytoskeleton of SKOV3 cells. First, we observed the morphological changes of the F-actin cytoskeleton by FITC-conjugated phalloidin and PI immunofluorescence staining. As shown in Fig. 10, SKOV3 cells in the control group displayed normal actin structure, single intact nucleus, and short F-actin bundles arranged around the cell membrane. After treated by CQDs/Cu_2_O, a large number of fluorescence spots were scattered throughout the cytoplasm, gradually disappeared as the CQDs/Cu_2_O concentration increased and the fluorescence intensity decreased, which significantly differed from that in normal F-actins. CQDs/Cu_2_O might therefore exhibit enhanced damage and disruption of F-actin. We also evaluated the content of F-actin cytoskeleton after treated by CQDs/Cu_2_O. The results of western blot demonstrates that the expression of F-actin in SKOV3 cells treated with CQDs/Cu_2_O significantly was significantly lower than that in the control group (Fig. 8d). It has been reported that some nanoparticles exhibit depolymerization effects or disrupt the cytoskeletal architecture and affect cell division, such as GO nanosheets [24], ZnO nanoparticles [25], gold nanoparticles [27], silver nanoparticles [27], and MOFs (IRMOF-3) [28]. Compared to the above nanoparticles and agents, we could demonstrate the disruption of the cytoskeleton filament owing to CQDs/Cu_2_O exposure and prove that the expression of the F-actin cytoskeleton decreased at the protein level. Hence, the suppression of SKOV3 migration by CQDs/Cu_2_O may be related to the downregulation of F-actin. It may be unique perspective and strategy in anticancer therapy.

## Conclusions

In summary, we demonstrated for the first time that CQDs/Cu_2_O composite displayed a greater sensitivity in SKOV3 cells than HeLa, A549, HT-29, HCT116 cancer cells and normal cells, such as BABL-3T3, HEK293T and J774A1, where the IC_50_ value of CQDs/Cu_2_O against SKOV3 cells was approximately threefold lower than other tested cancer cells and approximately 12-fold lower than normal cells. Amazingly, the IC_50_ of CQDs/Cu_2_O was approximately 114-fold and 75-fold lower than the IC_50_ of commercial artesunate (ART) and oxaliplatin (OXA), indicating CQDs/Cu_2_O possess strong antitumor activity than chemotherapeutics OXA and ART. This should be one of the most fascinating features of CQDs/Cu_2_O.

Interestingly, CQDs/Cu_2_O suppressed migration and angiogenesis processes in vitro mainly through downregulation the expression of VEGFR2 and MMP-2/9, which may be one of the main reasons for the selective inhibition tumor, since angiogenesis is an effective tumor treatment strategy to provide oxygen and nutrients for tumour progression and metastasis. CQDs/Cu_2_O also exhibited stronger antiangiogenic effects than commercial antiangiogenic inhibitor (SU5416). Furthermore, CQDs/Cu_2_O induces alterations in the cytoskeleton of SKOV3 cells by disruption of F-actin cytoskeleton which is critical for numerous physical cellular processes, including cell adhesion, migration, division, and cancer cell apoptosis. It is worth noting that CQDs/Cu_2_O also regulated angiogenesis-related genes in SKOV3 cells, such as Maspin and TSP1 gene, to suppress angiogenesis. This is first time to elucidate selectively mediation of CQDs/Cu_2_O in SKOV3 cells via transcriptome analysis, providing significant insights into the development and clinical application of CQDs/Cu_2_O.

From the above reasons, a probable mechanism for inhibition SKOV3 by CQDs/Cu_2_O has been proposed. CQDs/Cu_2_O selectively mediated of ovarian cancer SKOV3 cells death mainly through downregulation the expression of MMP-2, MMP-9, F-actin, and VEGFR2, meanwhile CQDs/Cu_2_O induced apoptosis of SKOV3 via S phase cell cycle arrest.

Our findings reveal a new role of CQDs/Cu_2_O in the selective and restrictive ovarian cancer SKOV3 cells. These findings could open a new avenue for using CQDs/Cu_2_O composite as potential therapeutics for cancer chemotherapy.

## Supplementary Information

**Additional file 1**: **Figure S1**. The enriched GO terms in the DEGs of cells treated by CQDs/Cu2O (3.12, 12.50 μg mL-1). The green, red, and blue bars represent the terms of biological process, cellular component, and molecular function, respectively. **Figure S2**. The viability of HUVEC cells after treated with CQDs/Cu2O by the MTT assay for 12 h.
