# Supplementary material for: Selective mediation of ovarian cancer SKOV3 cells death by pristine carbon quantum dots/Cu2O composite through targeting matrix metalloproteinases, angiogenic cytokines and cytoskeleton
Source: J Nanobiotechnology. 2021 Mar 4;19:68. doi: 10.1186/s12951-021-00813-8 (PMC7934478; doi:10.1186/s12951-021-00813-8)
Supplement: Supplementary file 1 — Additional file 1: Figure S1. The enriched GO terms in the DEGs of cells treated by CQDs/Cu2O (3.12, 12.50 μg mL-1). The green, red, and blue bars represent the terms of biological process, cellular component, and molecular function, respectively. Figure S2. The viability of HUVEC cells after treated with CQDs/Cu2O by the MTT assay for 12 h. [file 12951_2021_813_MOESM1_ESM.docx]

Supporting Information

**Selective mediation of ovarian cancer SKOV3 cells death by pristine carbon quantum dots/Cu_2_O** **composite through targeting matrix metalloproteinases, angiogenic cytokines and cytoskeleton**

Daomei Chen ^a, b^, Bin Li ^b^*, Tao Lei ^a-c^, Di Na ^a-c^, Minfang Nie ^a-c^, Yepeng Yang ^a-c^, Congjia, Xie ^a-c^, Zijuan He ^a-c^, Jiaqiang Wang ^a-c^*

a. National Center for International Research on Photoelectric and Energy Materials, School of Materials and Energy, Yunnan University, Kunming 650091, P.R. China. E-mail: jqwang@ynu.edu.cn

b. Key Laboratory of Medicinal Chemistry for Natural Resource, Ministry of Education, Yunnan University, Kunming 650091, P.R. China. E-mail: libin36@ynu.edu.cn

c. School of Chemical Sciences & Technology, Yunnan University, Kunming 650091, P.R. China.


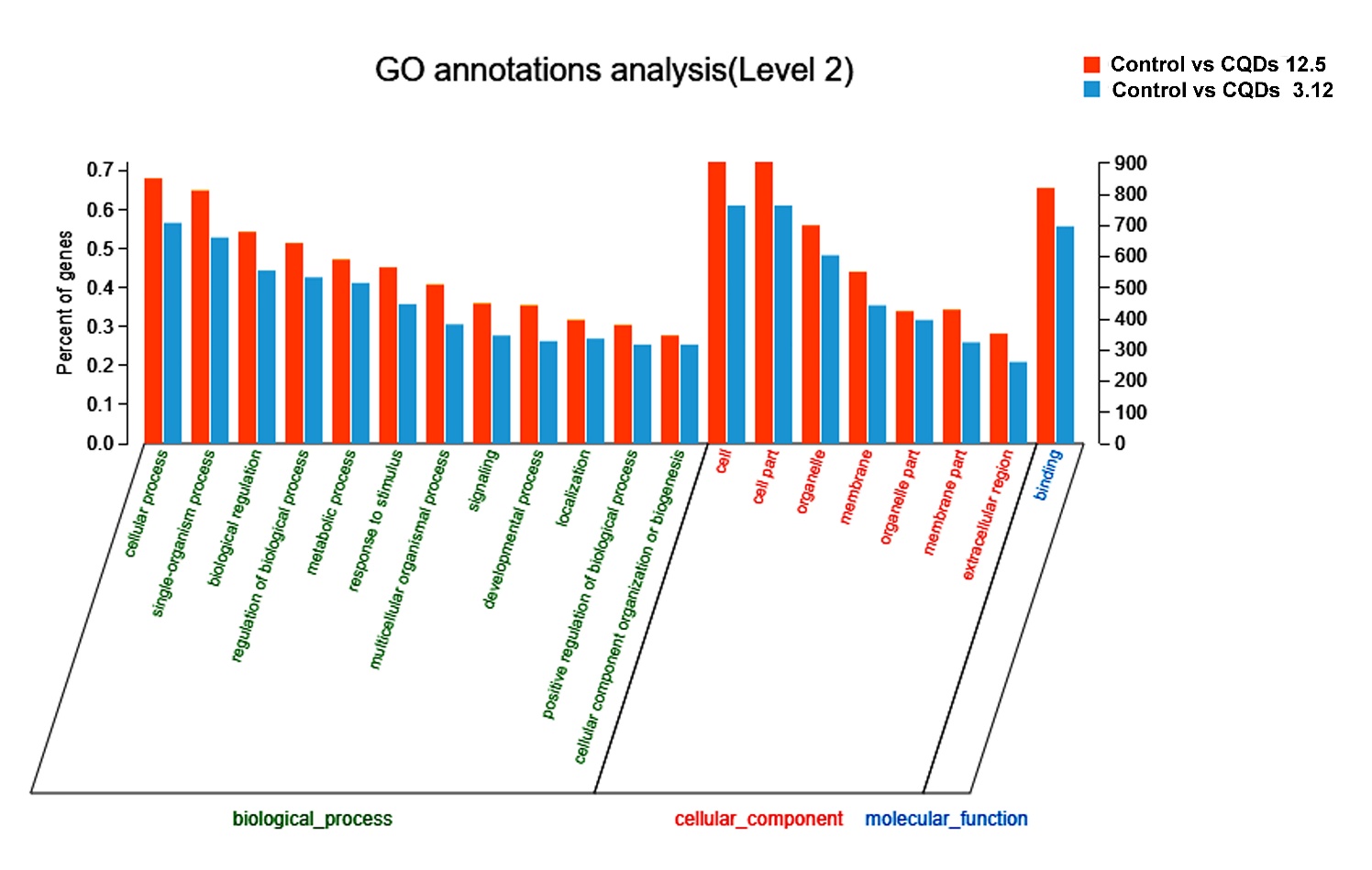


Figure S1. The enriched GO terms in the DEGs of cells treated by CQDs/Cu_2_O (3.12, 12.50 μg mL^-1^). The green, red, and blue bars represent the terms of biological process, cellular component, and molecular function, respectively.


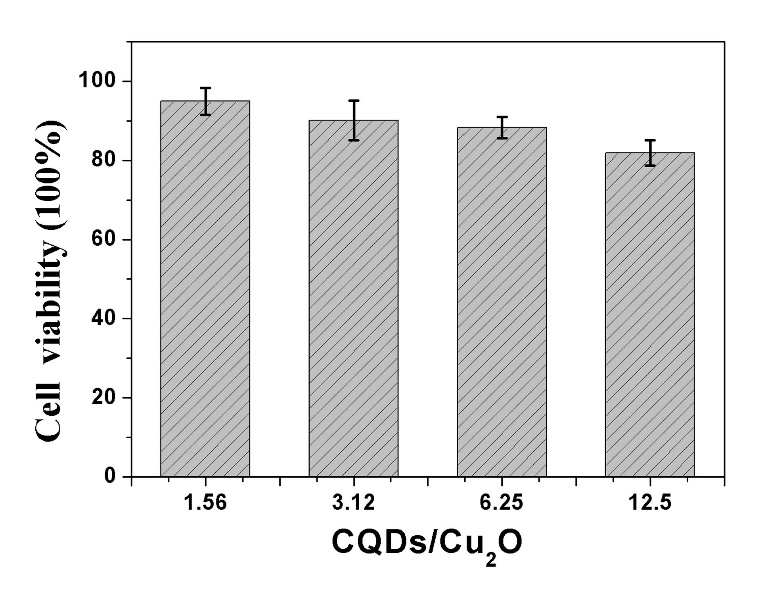


Figure S2 The viability of HUVEC cells after treated with CQDs/Cu_2_O by the MTT assay for 12 h.
